# Patient Views on Religious Institutional Health Care

**DOI:** 10.1001/jamanetworkopen.2019.17008

**Published:** 2019-12-27

**Authors:** Maryam Guiahi, Patricia E. Helbin, Stephanie B. Teal, Debra Stulberg, Jeanelle Sheeder

**Affiliations:** 1 Department of Obstetrics and Gynecology, University of Colorado School of Medicine, Aurora; 2 University of Colorado, Boulder; 3 Department of Family Medicine, The University of Chicago, Chicago, Illinois

## Abstract

**Importance:**

Federal protections allow health facilities to limit options to patients on the basis of religious values. Little is known about whether US adults consider religious affiliation when selecting facilities and whether they agree with such limitations.

**Objective:**

To understand patient views on religious institutional care.

**Design, Setting, and Participants:**

This is a population-based, cross-sectional survey study of US adults recruited from the probability-based AmeriSpeak Omnibus panel available from NORC (formerly the National Opinion Research Center) at the University of Chicago. Surveys were administered via internet or telephone during a 3-day fielding period in November 2017. Data analysis was performed from January 2018 to October 2019.

**Exposures:**

Participant characteristics, including religiosity measures.

**Main Outcomes and Measures:**

Whether patients consider religious affiliation when selecting a health care facility and their views on whether an institution’s religious values should take precedence over their health choices. Responses were compared by gender. When gender differences existed, regression models were performed, and thematic content analysis was applied to open-ended responses.

**Results:**

There were 1446 participants (745 [51.5%] male; mean [SD] age, 46 [17] years), for a survey completion rate of 24.5% and weighted cumulative response weight of 7.3%. Most respondents (62.6%) were white, and the most common religion was Protestant (28.2%). When specifically asked, only 6.4% reported that they considered religious affiliation when selecting a health care facility; most participants (71.3%) reported when selecting a health care facility that they did not care whether it is religiously affiliated, 13.4% preferred a religious affiliation, and 15.3% preferred no religious affiliation. There were no gender differences. Most participants (71.4%) believed that their health choices should take priority over an institution’s religious affiliation in services offered, and this was more common for women than for men (74.9% vs 68.1%; difference, 6.8%; 95% CI, 5.6%-8.2%; *P* = .005), who more commonly endorsed concerns for personal choice and autonomy over one’s own body.

**Conclusions and Relevance:**

This study suggests that most patients value their personal choices, yet do not consider an institution’s religious affiliation when choosing their source of health care. Women placed greater emphasis on their autonomy in comparison with an institution’s right to invoke religious restrictions to care. Given the growth in ownership of health care facilities by religious entities in the United States and increasing attention to conscientious objections, these findings point to a need for advocacy and legislation that effectively balances protections for religious institutions with protections for patients.

## Key Points

**Question:**

How do patients view and consider religious institutional health care?

**Findings:**

In a population-based survey study of 1446 US adults, only 6.4% reported that they considered religious affiliation when selecting a health care facility. Most patients (71.4%), particularly women, believed that their personal choices about their health should take priority over a facility’s religious values.

**Meaning:**

Although most patients believed that their health care choices should take precedence over a health care facility’s religious affiliation, the vast majority did not take religious affiliation into consideration when choosing where to receive care.

## Teaser

This population-based survey study examines patient views on religious institutional care, specifically whether they consider religious affiliation when selecting a health care facility and whether they believe that their personal choices about their health should take priority over a facility’s religious values.

## Introduction

The nature of US health care is shifting, in part because of the growing religious ownership sector. As of 2016, 18.5% of hospitals were religiously affiliated: 9.4% were Catholic-owned nonprofit hospitals, 5.1% were Catholic-affiliated hospitals, and 4.0% were other religious nonprofit hospitals.^1^ Catholic hospitals in particular have demonstrated significant growth recently; between 2001 and 2016, the number of acute care hospitals that were Catholic owned or affiliated grew by 22%, while the overall number of acute care hospitals decreased by 6% and the number of other nonprofit religious hospitals decreased by 38.3%.^1^ In 2016, 10 of the top 25 health care systems were Catholic sponsored.^1^ Almost half (46%) of all US Catholic hospitals are located in the Midwest,^2^ and 46 Catholic hospitals are designated as sole community hospitals because of their remote location from other major medical centers.^1^

Attendance at religious health care facilities can affect a patient’s access to services because of religious interpretations about care designated by the institution. Specific to Catholic health care facilities, clinicians are expected to abide by the *Ethical and Religious Directives for Catholic Health Care Services*,^3^ which places limitations on reproductive and end-of-life-care methods on the basis of the church’s moral teachings. Prior evidence^4^ has highlighted restrictions to care in Catholic facilities specific to contraception, sterilization, miscarriage management, and abortion. Recent media reports have highlighted conflicts in care with respect to transgender health and medical aid in dying. In other non-Catholic religious hospitals, contraceptive and sterilization services are generally provided, whereas abortion care is often restricted.^5^

In 1973, the US federal government provided the first protection to health care entities, known as the Church Amendment,^6^ to allow institutions to refuse to provide services that conflict with their religious beliefs or moral values. Such protections allow religious refusals of care to be implemented at the institutional level, without formally establishing religious doctrine as the basis of health care. Since that time, other conscience protections have emerged. In January 2018, the Trump administration founded the Conscience and Religious Freedom Division in the US Department of Health and Human Service Office for Civil Rights, to “protect the fundamental and unalienable rights of conscience and religious freedom.”^7^ The Office for Civil Rights issued a final conscience rule^7^ in May 2019 that broadens and enforces the right of religious health care entities—hospitals, clinics, insurance companies, and others—to invoke their institutional conscience to restrict options. In November 2019, a federal judge voided this rule, writing that the “stated justification for undertaking rule making in the first place—a purported ‘significant increase’ in civilian complaints relating to the conscience provisions—was factually untrue.”^8^

It is unknown to what extent patients consider religious affiliation when selecting their health care facilities and whether they believe institutions have the right of conscience over their own medical desires and needs. Prior surveys^9,10,11,12,13^ of factors that patients consider when choosing health care facilities have demonstrated that financial factors, travel distance and time, and hospital size were most important; none specifically inquired about religious affiliation. Given the growing religious health care sector in the United States, we set out to better understand how US patients perceive religious affiliations of health care organizations. We were also interested in understanding patient values as they relate to the medical care they receive within religious facilities. We hypothesized that women would endorse greater concerns about religious health care because of their greater awareness of and increased experiences with reproductive care restrictions.^4,14^

## Methods

We created a national cross-sectional survey of US adults that was administered by NORC (formerly the National Opinion Research Center) at the University of Chicago in November 2017. We used the AmeriSpeak Omnibus panel, which is a US multiclient survey that is derived from NORC’s National Sample Frame, is representative of more than 99% of US households, and includes additional coverage of difficult-to-survey population segments, such as rural and low-income households.^15^ The omnibus service ensures responses from at least 1000 adults aged 18 years and older after fielding the survey during a 3-day period (Friday through Sunday).^15^ Panel members receive a survey request that is not specific to the survey topic and are given the option to complete online or by telephone.^15^

All data were deidentified, and the Colorado Multiple Institutional Review Board deemed this study exempt with waiver of consent granted. This study follows the American Association for Public Opinion Research (AAPOR) reporting guideline.^16^

We focused survey development on understanding patient views of religious institutional health care. Our survey was informed by prior surveys,^9,10,11,12,13^ and questions were added or modified to extend queries related to religious institutional health care affiliations. We used expert panel review and piloted the preliminary draft with 5 lay individuals to ensure readability and absence of ambiguity. We first asked participants to select any and all considerations when selecting a health care facility and subsequently asked them to rate the most important consideration. Next, we specifically asked their preference about whether the health care facility they attend has a religious affiliation with the option to respond, “I do not care whether the hospital or clinic is or is not religiously affiliated,” “I prefer that the hospital or clinic is religiously affiliated,” or “I prefer that the health care institution is not religiously affiliated.” If they selected either of the latter 2 options, we used branching logic and asked “why?” with a free-text response option. Because we were interested in understanding how patients consider the implications of institutional conscience, we asked the following 2 questions: (1) “Do you think your personal choices about your health should have priority over the health care facility’s religious beliefs?” and (2) “Do you think an institution’s religious affiliation should take priority over an individual’s personal beliefs about their health care?” We followed each response with an open-ended “why” question. We ended our survey with participant characteristic queries available through the AmeriSpeak service, including ones specific to religiosity available on request, and made final revisions on the basis of AmeriSpeak’s suggestions.

We used SPSS statistical software version 25 (IBM) to calculate descriptive frequencies and compare gender responses using 2-sided χ^2^ analyses. If significant gender differences existed (with significance set at *P* ≤ .05), we sought to investigate independent factors associated with the survey query. To do so, we calculated separate logistic regression models for male and female participants including all relevant variables and reported adjusted odds ratios (aORs) and 95% CIs. We calculated survey weights according to gender and region using US Census data^17^ and applied them to our analyses. We also compared characteristics of responders with those of nonresponders. For open-ended queries, we used inductive thematic coding to categorize primary responses. The first coder created categories based on emerging themes and applied them to all responses. A second coder received the list of inductive codes and independently applied them to all the responses. The 2 coders then met to discuss any disagreements on categorizations, and final codes were assigned. Interrater reliability assessment was high (κ = 0.91).^18^ Data analysis was performed from January 2018 to October 2019.

## Results

A total of 1446 participants (745 men [51.5%]; mean [SD] age, 46 [17] years) completed the survey, for a survey completion rate of 24.5% and an American Association for Public Opinion Research weighted cumulative response rate of 7.3%. The overall margin of sampling error was ±3.6 percentage points at the 95% confidence level, including the design effect (Stefan Subias, AmeriSpeak representative, written communication, June 4, 2019). Survey responders were more likely than nonresponders to be older than 60 years (24.4% vs 21.1%; difference, 3.3%; 95% CI, 2.9%-3.8%; *P* = .008), to be white (62.6% vs 53.3%; difference, 9.3%; 95% CI, 8.6%-10.1%; *P* < .001), to be a college graduate (31.3% vs 20.1%; difference, 11.2%; 95% CI, 10.4%-12.0%; *P* < .001), and to live in the Midwest (28.2% vs 25.9%; difference, 2.3%; 95% CI, 2.0%-2.7%; *P* = .02). Nonresponders were more likely than responders to be aged 18 to 29 years (24.5% vs 19.0%; difference, 5.5%; 95% CI, 5.0%-6.1%; *P* < .001), to be black (16.8% vs 13.7%; difference, 3.1%; 95% CI, 2.7%-3.6%; *P* = .005), to be Hispanic (21.7% vs 15.3%; difference, 6.4%; 95% CI, 5.8%-7.0%; *P* < .001), to have an annual income less than $50 000 (56.6% vs 50.1%; difference, 6.5%; 95% CI, 5.9%-7.2%; *P* < .001), and to live in the South (30.1.% vs 27.1%; difference, 3.0%; 95% CI, 2.6%-3.5%; *P* = .03).

Participant characteristics analyzed by gender are demonstrated in Table 1. The majorities were men (51.5%), younger than 45 years (51.0%), non-Hispanic white (62.2%), employed (57.6%), living in a metropolitan area (88.8%), and completed the survey online (89.1%). The most common religion was Protestant (28.2%). Women were more likely than men to be younger than 45 years (54.0% vs 48.2%), be Hispanic (18.7% vs 12.1%), be black (15.8% vs 11.7%), have an annual income less than $50 000 (56.6% vs 44.0%), have at least 3 household members (52.4% vs 46.0%), have at least 1 household member younger than 18 years (40.2% vs 29.5%), identify as “just Christian” (23.4% vs 17.6%), and report they are very spiritual (32.4% vs 22.5%). Men were more likely than women to be college graduates (27.2% vs 35.0%), employed (52.4% vs 62.6%), married (43.7% vs 50.3%), and report their religion as either atheist, agnostic, or nothing in particular (20.4% vs 25.5%). When we applied survey weights to participant characteristics, there was no difference in the bivariate analyses.

**Table 1.** Survey Participant Characteristics Compared by Gender

Characteristic	Participants, No. (%)			*P* Value^a^
	All (N = 1446)	Women (n = 701)	Men (n = 745)	
Age, y				
18-29	275 (19.0)	146 (20.8)	129 (17.3)	.07
30-44	463 (32.0)	233 (33.2)	230 (30.9)	
45-59	355 (24.6)	170 (24.3)	185 (24.8)	
≥60	353 (24.4)	152 (21.7)	201 (27.0)	
Race/ethnicity				
Non-Hispanic				<.001
White	905 (62.6)	401 (57.2)	504 (67.7)	
Black	198 (13.7)	111 (15.8)	87 (11.7)	
Other^b^	26 (1.8)	18 (2.6)	8 (1.1)	
≥2 Races/ethnicities	47 (3.3)	24 (3.4)	23 (3.1)	
Asian	49 (3.4)	16 (2.3)	33 (4.4)	
Hispanic	221 (15.3)	131 (18.7)	90 (12.1)	
Education				
Less than high school	78 (5.4)	40 (5.7)	38 (5.1)	.02
High school graduate or equivalent	278 (19.2)	140 (20.0)	138 (18.5)	
Some college	638 (44.1)	330 (47.1)	308 (41.3)	
College graduate	452 (31.3)	191 (27.2)	261 (35.0)	
Employed (paid or self-employed)	833 (57.6)	367 (52.4)	466 (62.6)	<.001
Annual income, $US				
<25 000	354 (24.5)	199 (28.4)	155 (20.8)	<.001
25 000-49 999	371 (25.7)	198 (28.2)	173 (23.2)	
50 000-74 999	274 (18.9)	131 (18.7)	143 (19.2)	
75 000-99 999	193 (13.3)	81 (11.6)	112 (15.0)	
≥100 000	254 (17.6)	92 (13.1)	162 (21.7)	
Resides in metropolitan area	1284 (88.8)	630 (89.9)	654 (87.8)	.21
Region				
Northeast	228 (15.8)	112 (16.0)	116 (15.6)	.91
Midwest	408 (28.2)	193 (27.5)	215 (28.9)	
South	392 (27.1)	195 (27.8)	197 (26.4)	
West	418 (28.9)	201 (28.7)	217 (29.1)	
Married	681 (47.1)	306 (43.7)	375 (50.3)	.01
Household size ≥3 members	710 (49.1)	367 (52.4)	343 (46.0)	.02
≥ 1 Household member aged <18 y	502 (34.7)	282 (40.2)	220 (29.5)	<.001
Religion				
Roman Catholic	227 (15.8)	118 (17.0)	109 (14.8)	.01
Protestant	404 (28.2)	194 (27.9)	210 (28.5)	
Just Christian	293 (20.4)	163 (23.4)	130 (17.6)	
Atheist, agnostic, or nothing in particular	330 (23.0)	142 (20.4)	188 (25.5)	
Other	179 (12.5)	79 (11.4)	100 (13.6)	
Religiosity				
Not religious at all	370 (25.8)	161 (23.2)	209 (28.2)	.18
Slightly religious	364 (25.4)	181 (26.0)	183 (24.7)	
Moderately religious	503 (35.1)	252 (36.3)	251 (33.9)	
Very religious	198 (13.8)	101 (14.5)	97 (13.1)	
Attend religious services				
Never	327 (22.8)	141 (20.3)	186 (25.1)	.11
Less than monthly	618 (43.0)	305 (43.8)	313 (42.2)	
Monthly	151 (10.5)	82 (11.8)	69 (9.3)	
Almost once per week or more	341 (23.7)	168 (24.1)	173 (23.3)	
Spirituality				
Not spiritual at all	171 (12.0)	73 (10.5)	98 (13.4)	<.001
Slightly spiritual	335 (23.5)	137 (19.7)	198 (27.0)	
Moderately spiritual	530 (37.2)	259 (37.3)	271 (37.0)	
Very spiritual	390 (27.3)	225 (32.4)	165 (22.5)	
Survey mode completed online	1288 (89.1)	632 (90.2)	656 (88.1)	.20

^a^ *P* value demonstrated comparison of gender responses using χ^2^ analyses.

^b^ Includes Native American, Alaska Native, and any other race/ethnicity that the participant specifies.

The Figure shows responses regarding factors patients consider when selecting health care facilities. The most common responses were whether their insurance was accepted (72.5%), clinician reputation (60.2%), and/or facility reputation (58.5%); only 6.4% reported that they consider the religious affiliation of the health care institution. When asked which consideration was the most important, 44.6% reported insurance coverage and only 1.1% selected religious affiliation (Table 2). Responses were similar across genders.

**Figure.**
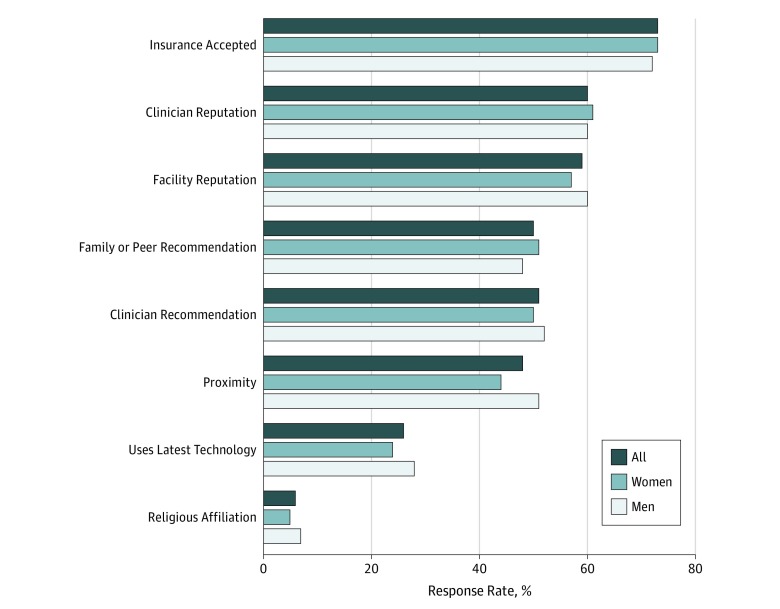
Health Care Facility Considerations by Gender Graph shows response rates to survey regarding factors considered when choosing a health care facility. The only statistically significant difference between women and men was for proximity of the facility (*P* = .01).

**Table 2.** Religious Health Care Beliefs Compared by Gender

Beliefs	Participants, No. (%)			*P* Value^a^
	All (N = 1446)	Women (n = 701)	Men (n = 745)	
Most important health care facility consideration				
Whether your insurance will be accepted	641 (44.6)	321 (46.1)	320 (43.2)	.43
Reputation				
Of clinician (eg, physician)	256 (17.8)	136 (19.5)	120 (16.2)	
Of facility	185 (12.9)	80 (11.5)	105 (14.2)	
Recommendations				
From health care professionals	158 (11.0)	72 (10.3)	86 (11.6)	
From friends or family	72 (5.0)	34 (4.9)	38 (5.1)	
Geographical proximity	63 (4.4)	30 (4.3)	33(4.5)	
Uses latest technology	35 (2.4)	13 (1.9)	22 (3.0)	
Religious affiliation of health care institution	16 (1.1)	7 (1.0)	9 (1.2)	
Other	11 (0.8)	4 (0.6)	7 (0.9)	
Consideration of religious affiliation when selecting a facility				
Do not consider	1023 (71.3)	494 (70.7)	529 (71.9)	.74
Prefer religious facility	193 (13.4)	99 (14.2)	97 (13.2)	
Prefer nonreligious facility	219 (15.3)	105 (15.0)	110 (14.9)	
Personal choices about health should have priority over the health care facility’s religious affiliation	1024 (71.4)	522 (74.9)	502 (68.1)	.005
Institution’s religious affiliation should take priority over personal health choices	249 (17.4)	108 (15.6)	141 (19.2)	.07

^a^ *P* value is calculated using χ^2^ analyses to compare gender responses.

When we specifically asked whether they prefer religious affiliation when selecting a health facility, most participants (71.3%) reported they do not care whether the facility is religiously affiliated, 13.4% prefer a religious affiliation, and 15.3% prefer no religious affiliation (Table 2); we did not detect differences by gender. Among the 193 respondents who preferred a religious affiliation, 143 (74.1%) provided comments. Thematic responses included desire for a facility with beliefs similar to those of the patient (26.6%); belief that care is better, safer, and/or more compassionate (23.8%); desire for a facility to involve faith and/or God (18.9%); belief that care is of higher ethical and/or moral standards (10.5%); preference to involve prayer and/or religious members (10.5%); personal preference (8.4%); and belief that religious facilities provide charity care (1.4%).

Among the 219 respondents who reported they preferred a nonreligious facility, 156 (71.2%) provided comments. Thematic reasons included concerns that care is biased or based on other concerns (28.2%), belief that religion should not interfere with health care (23.7%), belief that care is worse at religion institutions because it is not based on science (17.3%), concern about restrictions to care (16.0%), and general concerns about religion (14.7%).

Most respondents (71.4%) agreed with the statement that their personal choices about their health should have priority over a health care facility’s religious affiliation (Table 2), and this was more common for women compared with men (74.9% vs 68.1%; difference, 6.8%; 95% CI, 5.6%-8.2%; *P* = .005). Table 3 demonstrates multivariable analyses. Men who reported being nonreligious (aOR, 2.68; 95% CI, 1.61-4.47) and/or residing in the Northeast (aOR, 1.78; 95% CI, 1.10-3.05) or Midwest (aOR, 1.61; 95% CI, 1.02-2.56) were more likely to agree with this statement compared with those in the West (Table 3). Men who had at least 3 household members (aOR, 0.50; 95% CI, 0.32-0.79) and/or reported attending religious services at least once a week (aOR, 0.57; 95% CI, 0.37-0.88) were less likely to agree with this statement (Table 3). In comparison, no factors emerged as significant among women. Applying survey weights had no effect on our models.

**Table 3.** Multivariable Logistic Models for the Belief That Personal Health Choices Should Take Priority Over an Institution’s Religious Affiliation by Gender

Variable	aOR (95% CI)	
	Women	Men
Age, y		
18-29	0.82(0.43-1.57)	0.63 (0.34-1.17)
30-44	0.83 (0.45-1.54)	0.90 (0.52-1.55)
45-59	0.69 (0.39-1.22)	0.76 (0.46-1.26)
≥60	1 [Reference]	1 [Reference]
Black, non-Hispanic	1.35 (0.78-2.36)	1.22 (0.69-2.18)
Hispanic	0.92 (0.56-1.51)	0.89 (0.52-1.51)
College graduate	1.10 (0.72-1.70)	1.13 (0.77-1.66)
Employed (paid or self-employed)	1.29 (0.88-1.89)	0.88 (0.59-1.31)
Annual income <$50 000	1.56 (0.77-1.73)	1.23 (0.83-1.82)
Resides in metropolitan area	1.11 (0.62-1.99)	0.85 (0.50-1.46)
Region		
Northeast	0.78 (0.43-1.39)	1.78 (1.10-3.05)
Midwest	0.66 (0.40-1.11)	1.61 (1.02-2.56)
South	0.72 (0.43-1.21)	1.55 (0.98-2.46)
West	1 [Reference]	1 [Reference]
Married	1.11 (0.75-1.65)	0.75 (0.50-1.13)
Household size ≥3 members	1.11 (0.66-1.86)	0.50 (0.32-0.79)
≥ 1 Household member aged <18 y	0.76 (0.45-1.28)	1.47 (0.89-2.41)
Roman Catholic	0.73 (0.45-1.18)	0.74 (0.46-1.18)
Atheist, agnostic, or nothing in particular	1.49 (0.84-2.64)	2.68 (1.61-4.47)
Very or moderately religious	0.82 (0.52-1.30)	0.72 (0.46-1.13)
Attends religious services almost once a week or more	0.91 (0.59-1.40)	0.57 (0.37-0.88)
Very or moderately spiritual	0.98 (0.61-1.58)	1.24 (0.81-1.89)

Abbreviation: aOR, adjusted odds ratio.

Among the 1024 respondents who believe that their health choices should take priority over an institution’s religious affiliation, 865 provided comments. Thematic responses included reference to personal choice and/or autonomy over one’s own body (60.6%), that an institution’s role should be focused on health over religion (24.0%), that an institution’s religious affiliation may not be aligned with a patient’s beliefs (8.0%), that the patient is the one to bear financial responsibility (3.9%), that a religious institution may be the only close or available facility and should not dictate care according to religion (1.2%), and that religious institutions receive government funding and so should not direct care according to religion (0.9%). Women were more likely than men to vocalize concerns over personal choice and/or autonomy over one’s own body (64.3% vs 56.9%; difference, 7.4%; 95% CI, 5.8%-9.3%; *P* = .03).

Fewer respondents (17.4%) agreed that an institution’s religious affiliation should take priority over their personal health choices (Table 2), and this did not differ by gender (15.6% for women vs 19.2% for men; difference, 3.6%; 95% CI, 2.7%-4.7%; *P* = .07). Among the 294 respondents who agreed with this query, 186 provided comments. Thematic responses included that patients have the option to go to another facility (45.2%), reference to institutional religious freedom (38.7%), a private facility has the right to religious freedom (5.9%), preference for religious care (4.8%), belief that health concerns will be prioritized over religion (4.2%), and personal preference (1.1%).

## Discussion

This national cross-sectional survey of 1446 US patients provides insights about religious medical care considerations. Even when we prompted participants by specifically inquiring about religious affiliation, there were small proportions that had preferences either for (13.4%) or against (15.3%) attendance at a religiously affiliated health care facility. In contrast, when we inquired about the implications of religious institutional restrictions to care with respect to potential services offered, the majority favored their personal autonomy over the religious values of their health care facility. This discordance between how most patients choose health facilities and their beliefs about how they should receive care suggests a general lack of understanding specific to the notion of institutional conscience and may serve as the basis for conflicts in care.

Being a woman in and of itself was associated with support for personal autonomy over institutional conscience. This likely reflects that religious restrictions to care are of greater concern for women because of reproductive care restrictions and explains why so many commented about concerns for personal choice and/or autonomy over one’s own body.^4,14^ Prior studies have highlighted restrictions to women’s health services in Catholic facilities^4^ and have demonstrated that patients often do not realize when their hospital is Catholic^19^ and that many do not realize the extent to which Catholic doctrine affects provision of reproductive health care services.^20,21^ Lack of transparency by Catholic health facilities is a contributing factor^14^; a recent website analysis^2^ of all Catholic hospitals in the United States found that less than one-third provided any description of restrictions to care, and a national mystery caller survey^22^ highlighted how patients are often not informed of service restrictions when placing birth control appointments.

We were surprised that there were few other factors associated with the support for personal autonomy over institutional conscience among men. Not surprisingly, we found that men who do not associate with a religion were more likely to value personal autonomy over institutional conscience. In contrast, those who reported frequent attendance at a religious facility and/or a higher number of household members were less likely to share those values. The finding that men in the Midwest were more likely than those in the West to value their autonomy may reflect the higher proportion of Catholic hospitals in this region and greater knowledge about religious restrictions to care.^2^ We anticipated that age may have played a role because younger adults tend to be less religious. Because our survey panel oversampled older adults compared with younger ones, it is possible that we may have missed differences between age groups. Recent lawsuits^23^ have highlighted emerging cases of conflict in Catholic settings with respect to end-of-life care given expanding legislation and support for medical aid in dying. Such conflicts in care may be of greater relevance to men in the future, particularly older ones, and underscores the need for transparency and greater awareness about religious restrictions to care as Catholic health care systems continue to expand and affect a growing proportion of patients.

Strengths of our study include that we specifically targeted concerns for religious health care, analyzed religiosity measures, and provided qualitative themes. Importantly, AmeriSpeak uses probability-based panels, which are becoming the standard given that traditional household surveys are less feasible and tend to differentially exclude many people.^15^

### Limitations

 Our study also has limitations. Although we had a diverse sample, we still oversampled white individuals with higher education and higher income, and so our findings may be less representative of the views and opinions of underrepresented groups, including black and Hispanic populations. Our cumulative survey response rate was also low (7.3%), which is accounted for, in part, by the way in which AmeriSpeak obtains their panels and response rates.^16^ Unlike other surveys that often remove panel members if they do not respond to surveys when calculating response rates, AmeriSpeak includes panel members who do not respond. In addition, the omnibus survey service has a short fielding period of only 3 days. Although there may be concerns for responder bias based on the subject of our survey, participants were not informed of this before survey initiation.

## Conclusions

Our findings demonstrate that most patients place great emphasis on their autonomy, effectively disagreeing with ongoing protections for institutions to restrict care on the basis of their religious or moral values. Advocacy efforts are needed to enact legislation that counterbalances protections for institutions with protections for patients. Because women are disproportionately affected by religious restrictions to care, as are LGBTQIA (lesbian, gay, bisexual, transgender, queer, intersex, and asexual) patients and those in rural settings,^24^ advocates must work toward antidiscriminatory policies and legislation. In Washington state, legislation has passed that enforces all hospitals to report restrictions to care on their websites.^25^ Because some patients in religious settings may not have other reasonable or viable options for health care access and/or may be faced with life-threatening conditions or need medically indicated care, stronger emergency care protections are urgently needed. Broader consideration should also be given for protections that ensure provision of medically indicated care, even in nonemergent settings.
